# Supplementary material for: CLIC1 Protein Accumulates in Circulating Monocyte Membrane during Neurodegeneration
Source: Int J Mol Sci. 2020 Feb 21;21(4):1484. doi: 10.3390/ijms21041484 (PMC7073046; doi:10.3390/ijms21041484)
Supplement: Supplementary file 1 [file ijms-21-01484-s001.pdf]

Supplementary

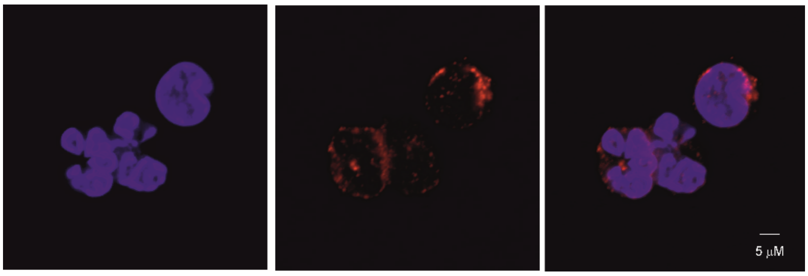

Figure S1. CLIC1 presence in PBMCs. Confocal imagine of whole CLIC1 protein immunostaining in two representative lymphocytes and one monocyte. The two cell types can be easily recognized based on the shape of nucleus (blue): in lymphocytes the nucleus is polylobate, in monocytes the nucleus is compact and occupies most of the cytoplasm. CLIC1 protein staining intensity (red) detected in monocytes is higher than fluorescence observed in lymphocytes.

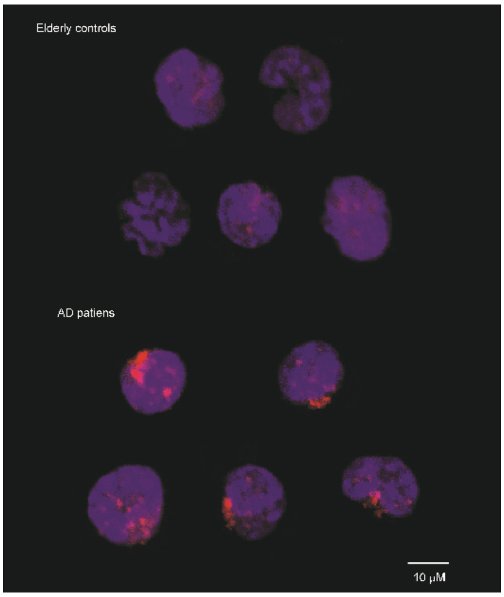

Figure S2. CLIC1 protein increases in isolated monocytes from AD patients. Examples of CLIC 1 immunostaining (red) in monocytes obtained from blood samples of 5 different healthy elderly individuals and 5 AD patients.

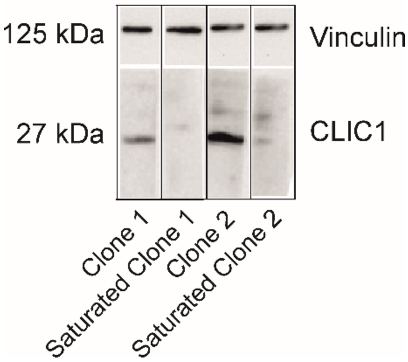

Figure S3. Polyclonal amino-terminus CLIC1 antibody specificity. Western blot analysis of protein lysates incubated with AB-NH2 candidate clones (5 μg/ml) pure or saturated with the target peptide. Saturated clones were obtained by incubation with 10-fold peptide concentration. Antibody specificity was demonstrated by the presence of a unique and intense band corresponding to CLIC1 molecular weight (27 kDa) and the absence of bands in the presence of saturated antibody. Although Clone 2 shows a more intense band, we selected Clone 1 because its saturated control is accompanied by absence of any signal.

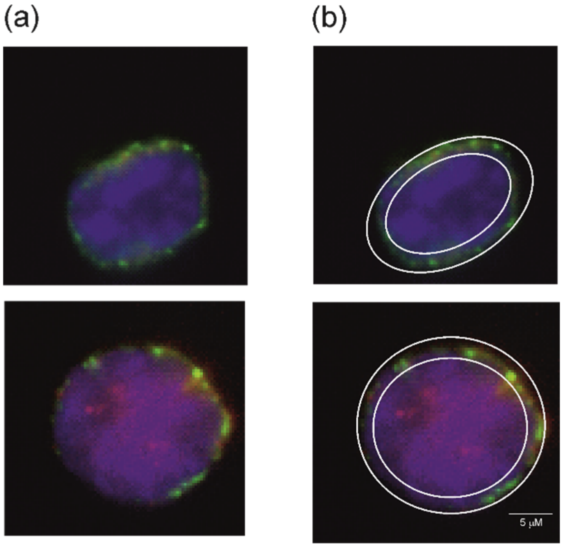

Figure S4. Procedure to measure fluorescence level localized to the membrane of isolated monocytes. (a) Single cell images of monocyte stained with the polyclonal antibody against the amino terminus of CLIC1 protein. (b) Concentric ROIs are placed by eye on each cell and only the fluorescence within the two lines were measured.

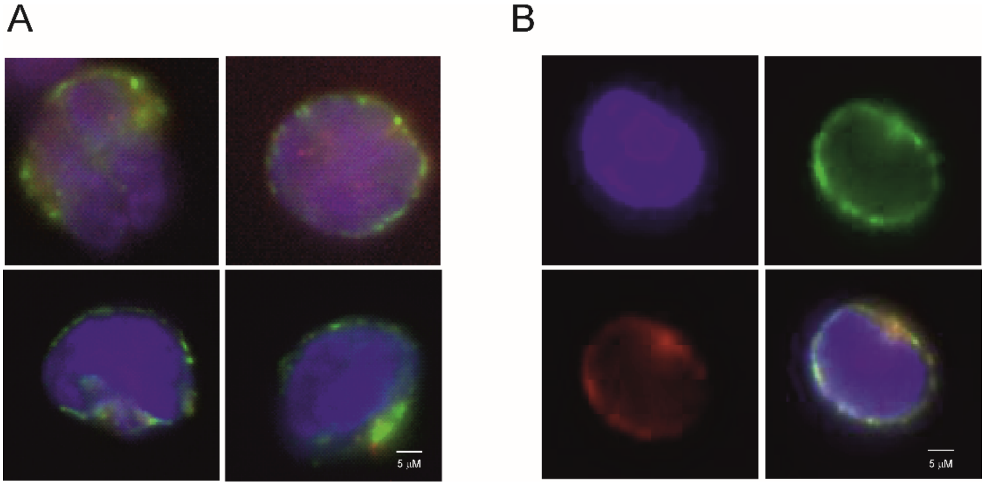

Figure S5. A: examples of CLIC1 protein membrane localization in monocytes isolated from AD patients' blood. B: Upper left picture shows cell nucleus stained with DAPI. In upper right, live cells were primary stained with the AB-NH2 to selectively isolate membrane CLIC1 signal, preventing membrane disruption (green). In bottom left, permeabilized cells were stained with whole-CLIC1 antibody to visualize whole protein distribution (red). Bottom right figure shows the merge of the three channels.
